# The Role of IL-23 in the Pathogenesis and Therapy of Inflammatory Bowel Disease

**DOI:** 10.3390/ijms241210172

**Published:** 2023-06-15

**Authors:** Aleksandra Korta, Julia Kula, Krzysztof Gomułka

**Affiliations:** 1 Student Scientific Group of Adult Allergology, Wroclaw Medical University, 50-369 Wroclaw, Poland; aleksandra.korta@student.umw.edu.pl (A.K.); julia.kula@student.umw.edu.pl (J.K.); 2 Clinical Department of Internal Medicine, Pneumology and Allergology, Wroclaw Medical University, 50-369 Wroclaw, Poland

**Keywords:** interleukin-23, inflammatory bowel disease, Crohn’s disease, ulcerative colitis, pathogenesis, 12/23 cytokines, effectors, treatment

## Abstract

Interleukin-23 (IL-23) is a proinflammatory cytokine produced mainly by macrophages and antigen-presenting cells (APCs) after antigenic stimulation. IL-23 plays a significant role as a mediator of tissue damage. Indeed, the irregularities in IL-23 and its receptor signaling have been implicated in inflammatory bowel disease. IL-23 interacts with both the innate and adaptive immune systems, and IL-23/Th17 appears to be involved in the development of chronic intestinal inflammation. The IL-23/Th17 axis may be a critical driver of this chronic inflammation. This review summarizes the main aspects of IL-23’s biological function, cytokines that control cytokine production, effectors of the IL-23 response, and the molecular mechanisms associated with IBD pathogenesis. Although IL-23 modulates and impacts the development, course, and recurrence of the inflammatory response, the etiology and pathophysiology of IBD are not completely understood, but mechanism research shows huge potential for clinical applications as therapeutic targets in IBD treatment.

## 1. Introduction

**Figure 1:**
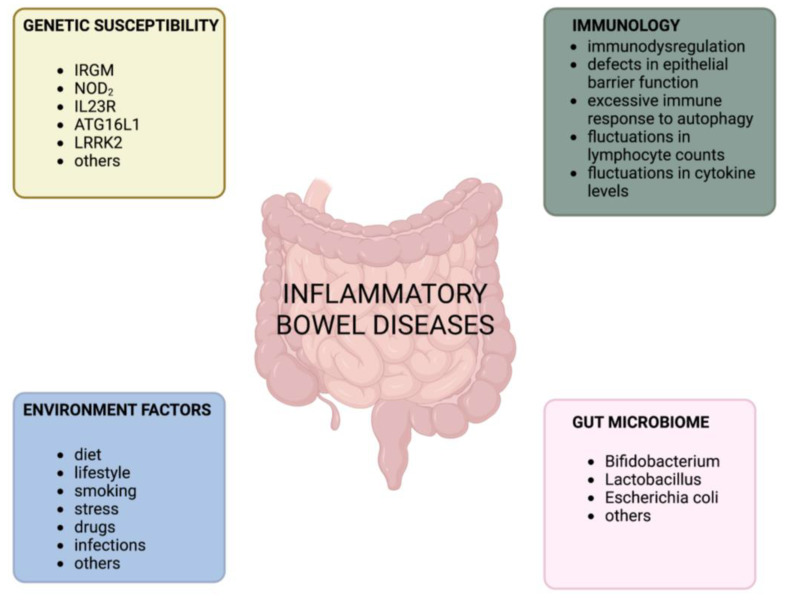
Key factors involved in the pathogenesis of inflammatory bowel disease.

The gastrointestinal tract plays a crucial role in maintaining immune homeostasis [1]. It is responsible for balancing tolerance to commensal bacteria, food antigens, and the body’s own antigens while also defending against pathogens by inducing an inflammatory response [1]. When the balance between tolerance and inflammation in the gastrointestinal tract is disrupted, it can lead to inflammatory bowel disease (IBD) [1]. Crohn’s disease (CD) and ulcerative colitis (UC) are the two main clinical forms of IBD, each with distinct clinical features [2,3]. These chronic inflammatory disorders of the gastrointestinal tract manifest as lifelong relapsing and remitting phases [4,5] and are characterized by uncontrolled innate and adaptive immune responses that result in mucosal inflammation [6]. Although the etiology and pathophysiology of IBD are not fully understood, studies on various murine models have provided insight into its pathogenesis. Factors such as excessive and aberrant immune responses to pathogens, defects in gut barrier integrity, gut microbiota, environmental factors, genetic predispositions, and impaired regulation of the immune system have a significant impact on the development of IBD [7,8,9,10,11] (Figure 1).

Functional studies of different genomes have helped in exploring the host genetic susceptibility of loci in IBD patients [12]. The presence of links between pathogenetic mechanisms in CD and UC, which are consistent with the high number of genetic concordant loci between them [13], suggests that common therapies may be effective [14,15].

The incidence of IBD in the general population is on the rise, creating a challenge for diagnosis and treatment [16]. IBD has a multifactorial etiology, which makes it difficult to address. As treatment and diagnosis costs continue to increase, there is a growing interest in finding new effective treatments [17]. Collaboration between different specialists and clinical decision-makers is becoming more common. Cytokines mediate the composite interaction between innate and adaptive immune responses. Disorders in this transmission can initiate and maintain inflammation of the mucosa. Research has identified cytokine networks that play a crucial role in the induction and regulation of IBD, which opens up new opportunities for treatment. Interleukin (IL)-12 is a cytokine predominantly generated by cells in the innate immune system, which comprises four members: IL-12, IL-23, IL-27, and IL-35. Antigen-presenting cells (APCs) produce IL-12, IL-23, and IL-27 during antigen presentation to naïve T cells, but IL-35 is a product of regulatory T and B cells [18,19]. IL-12 cytokines are critical to regulating cellular pathways, maintaining intestinal homeostasis, and activating proinflammatory responses to protect against infection while also inhibiting uncontrolled immune responses that cause autoimmune disorders. IL-23, on the other hand, acts as a crucial mediator in tissue damage and the dysregulation of IL-23 and its receptor signaling, which are strongly implicated in a variety of inflammatory disorders [18,20,21]. IL-23 has been identified as a factor in the pathogenesis of autoimmune inflammatory diseases, including IBD and cancer. This review will focus on and discuss the crucial importance of the proinflammatory cytokine IL-23 in the pathogenesis of IBD.

## 2. Biological Function of IL-23

**Figure 2:**
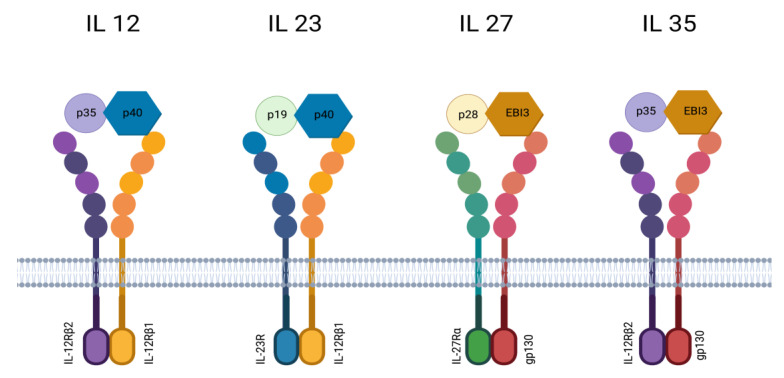
The family of interleukin 12 includes IL 12, IL 23, IL 27, and IL 35. Each one is made up of different subunits, including p19, p28, p35, p40, and EBI3. These subunits interact with various receptors and receptor chains.

IL-23 is a heterodimeric proinflammatory cytokine [22] that is a member of the IL-12 cytokine family, together with IL-12, IL-27, and IL-35 [23]. IL-27 and IL-35 have inhibitory properties, while IL-12 and IL-23 play roles in proinflammatory reactions [24]. The family members are different in the composition of their subunits: IL-23 is composed of an IL-12p40 subunit complexed with a unique IL-23p19 via a disulfide bond, while the closely related IL-12 is composed of a 12p40 subunit complexed with an IL-12p35 subunit [25]. IL-23 interacts with two receptor chains—the specific IL-23 receptor and IL-12Rβ1 (Figure 2). First of all, IL-23 is involved in the development of Th17 cells and their differentiation and proliferation. Except for Th17 cells, innate immune cells respond to IL-23–γδT cells, natural killer T (NKT) cells, and innate lymphoid cells (ILCs) [26,27].

## 3. Factors Controlling the Production of IL-23

### 3.1. Microbial Stimulation

The principal cellular sources of IL-23 are macrophages and antigen-presenting cells (APCs) [28]. The mode of activation of dendritic cells (DCs) critically affects the expression of IL-23 [29]. The response of macrophages and DCs to antigenic stimulation depends on a variety of factors that affect the potency of IL-23 production. Some of these factors are inherent to the cell, while others are related to the surrounding environment [30]. When exposed to microbial stimuli, DCs and macrophages increase their production of IL-23. The type of microbial stimulus and the specific pattern recognition receptors involved play a significant role in determining the production of IL-23 relative to other cytokines. Lamina propria CD14+ CD33+ macrophages are especially likely to produce IL-23 in the intestine when exposed to commensal bacteria such as *Escherichia coli* and *Enterococcus faecalis*. Moreover, it has been observed that exposure to *Escherichia coli* increases the expression of IL-12 and IL-27 [29]. The quantity of these bacteria is higher in patients with CD than in those without [31,32]. IL-23p19 expression is elevated in inflamed mucosa from patients diagnosed with both UC and CD. It is derived mainly from CD68+ macrophages and DCs [33]. The non-inflamed mucosa of the terminal ileum exhibit the highest level of IL-23 expression. CD11c+ DCs in the lamina propria of this region express the shared p40 subunit constitutively in the presence of intestinal microbiota [34]. The production of IL-23 by DCs and IL-17 by DCs and CD4+ T cells increases upon the stimulation of the NOD2 receptor, cytosolic nucleotide-binding oligomerization domain-containing protein 2 [30]. NOD2 disease-associated variants are predicted to enhance IL-23 responses [35]. In fact, these variants were found to augment p40 secretion from DCs when stimulated by adherent invasive *Escherichia coli*. Research conducted using activated DCs revealed that NOD2 variants linked to CD are also associated with the decreased expression of miR-29, a microRNA that can indirectly downregulate IL-23p19 through its effects on ATF2. Moreover, IL-23p19 production and Th17 responses in co-culture experiments were reduced by miR-29 expression in DCs [35]. Cellular stress can release extracellular adenosine nucleotides that accumulate in areas of inflammation [36]. Furthermore, extracellular adenosine augments the induction of IL-23p19 by dendritic cells via P2 purinergic receptor signaling in response to *Escherichia coli*. Simultaneously, it suppresses IL-12 responses in a reciprocal manner [29]. An additional layer of regulation in the IL-23 response is observed at the intracellular metabolism level within dendritic cells and macrophages [37]. Dietary components from external sources can directly affect the metabolic rewiring of immune cells, which controls the generation of IL-23 and autoimmunity. Research shows that several genes linked to IBD encode proteins importantly involved in metabolism [38,39]. This includes FAMIN, an enzyme that helps macrophages recycle purine nucleotides through its core catalytic activities, which is widely conserved throughout evolution [40]. The complex system of pro- and anti-inflammatory cytokines, as well as other molecules, affects the reaction of IL-23.

### 3.2. IL-1 Cytokine Network

The IL-10 released by a specific group of peripheral blood mononuclear cells (PBMCs) is a crucial factor in regulating IL-23p19 production via distinct CD14+ mononuclear cells through paracrine signaling [41]. IL-10R mutations associated with inflammatory bowel disease (IL-10 and IL-10 receptor mutations in very early-onset inflammatory bowel disease) lead to the loss of the inhibitory effect of IL-10 on proinflammatory cytokine production in patient cells. However, blocking IL-1R1 was effective in reducing the expression of IL-23p19 [41]. Scientists have found that the cytokines IL-1α/IL-1β and IL-10 play an important role in controlling monocytes that produce IL-23 through autocrine and paracrine sensing. Researchers identified a transcriptional signature of IL-23-producing monocytes that was able to predict disease and resistance to anti-TNF therapy. This signature was also able to differentiate between hyperinflammation-associated IL-23 production in patients with severe ulcerating active CD and homeostatic intestinal IL-23 production [41]. The findings indicate that IL-10-producing monocytes regulate IL-23-producing cells through a paracrine mechanism. IL-10 signaling serves as a negative feedback mechanism, while IL-1β acts as a positive regulator of IL-23 production. IFN-γ acts as an amplifier [42]. Research shows that IL-10 and IL-1 are key factors in regulating the production of IL-23 in monocytes. Scientists distinguish between the homeostatic production of IL-23 and the production of IL-23 associated with hyperinflammation in patients with severe ulcerating active Crohn’s disease and non-responsiveness to anti-TNF treatments. Overall, there are subgroups of patients with IBD who may benefit from therapies that target IL-23p19 and/or IL-1α/IL-1β upstream of IL-23 [41].

### 3.3. The cAMP Pathway

It has been demonstrated that ATP increases IL-23 expression while simultaneously suppressing IL-27 expression. ATP selectively affects the expression profile of IL-12-related cytokines, altering the ratios of IL-12/IL-23 and IL-27/IL-23 in favor of IL-23 [29]. Moreover, it has been reported that monocyte-derived dendritic cells (MoDCs) that have been activated with combinations of cytokines, CD40L, and TLR ligands, and intact bacteria express IL-23 and IL-27 subunits [28,43,44,45,46]. These findings indicate that ATP mediates the reciprocal regulation of IL-12/IL-27 and IL-23 through two distinct P2 receptors. This process is also induced by prostaglandin E2 via cyclic adenosine monophosphate (cAMP)-elevating EP2/EP4 receptors. IL-23p19 expression can be enhanced by PGE2. As IL-23 seems to play a crucial role in T cell memory generation and autoimmunity, P2 receptors may represent a hopeful target for the creation of novel treatment methods for infections, autoimmune diseases, and cancer [29]. When macrophages and DCs are stimulated with antigens, such as Toll-like receptor (TLR) agonists, intracellular signaling cascades are initiated. These cascades intensify the transcription of the IL-23A gene and the secretion of its protein [47]. Furthermore, the TLR4 agonist lipopolysaccharide (LPS) has the ability to increase both IL-12 and IL-23 levels, particularly when IFNγ is present [48,49]. Monocyte-derived dendritic cells (MoDCs) can produce IL-23 in response to both Gram-negative and Gram-positive bacteria. However, this response requires the bacteria to be intact. This suggests that dendritic cell activation via bacteria differs from dendritic cell activation via TLR ligands.

### 3.4. CXCL8 Chemokine Family

Neutrophils that infiltrate the tissue also serve as a source of IL-23 in the inflamed colon [50]. Neutrophils that infiltrate tissue express CXCR1 and CXCR2, which are receptors for the CXCL8 chemokine family [51] and are considered the main source of IL-23 in the colons of patients with IBD. In addition, IL-23-producing CXCR1+ CXCR2+ neutrophils circulating in blood samples were identified in patients with IBD, as well as in non-IBD patients and healthy adult donors [50]. Tissue-infiltrating neutrophils were found to be the primary source of IL-23 in the colons of pediatric patients with IBD. IL-23(+) human leukocyte antigen-DR(+) or IL-23(+)CD14(+) cells were either barely detectable or undetectable. The blood samples of both patients with IBD and healthy individuals consist of colonic IL-23(+) neutrophils expressing the C-X-C motifs (CXC)R1 and CXCR2, receptors for the CXC ligand 8 (CXCL8) chemokine family, and a corresponding CXCR1(+)CXCR2(+)IL-23(+)subpopulation of neutrophils, although CXCL8-family chemokines were only found to be elevated in the colon tissue of patients with IBD [50]. IL-23 receptor expression requires the transcriptional activity of retinoic acid-related orphan receptor γt (RORγt). Studies have shown that, when TGF-β, IL-6, and IL-1 are all present, RORγt is activated, and this, in turn, induces the expression of the IL-23 receptor, allowing for additional IL-23 signaling [52,53].

## 4. Effectors of the IL-23 Response

### 4.1. The IL-23-Th17 Axis

IL-23 acts on members of the innate immune system and enables the growth and maintenance of Th17 cells. IL-23 does not induce Th17 differentiation or IL-17A production from naïve CD4+ T cells by itself, as these cells lack significant expressions of IL-23R [28], although it appears that IL-23 plays a part in their ongoing survival and growth [52]. CD4+ T cells that do not express the IL-23 receptor develop into Th17 cells after receiving signals from antigen-presenting cells and cytokines such as IL-6, IL-21, and TGF-β. This leads to the mutual up- and downregulation of the signature RORγt and Forkhead box P3 (FoxP3) transcription factors, as well as an increase in IL-23 receptor expression. IL-23 signaling through its corresponding receptor supports the survival and growth of Th17 cells that produce IL-17 and IL-22. Variants in IL-23R that provide protection against autoimmune disease reduce this effect. Upon reactivation, Th17 cells can transform into a Th1-like phenotype, which is characterized by the expression of T-bet and the production of the colitogenic cytokine IFN-γ, especially when IL-23/IL-12 levels are high [30]. IL-23 contributes to inflammation in the intestine by affecting both the innate and adaptive immune cells in various ways. IL-23 boosts the formation and longevity of Th17 cells, leading to the production of cytokines, i.e., IL-17, IFNγ, IL-22, and granulocyte–macrophage-colony-stimulating factor (GM-CSF) [54,55]. The latter promotes the accumulation of granulocyte-monocyte progenitors (GMPs) and activated eosinophils within the intestine. IL-23 prevents the development of regulatory T cells and promotes the generation of cytokines, such as IL-17, IL-22, and GM-CSF, from group 3 innate lymphoid cells (ILC3s). The autocrine effects of IL-23 on macrophages have been reported, including the production of proinflammatory cytokines and the recycling of IL-23R. IL-23 also causes intestinal epithelial cells to produce regenerating (Reg) proteins, which attract neutrophils and act as another source of IL-22. IL-22 has both positive and negative effects on intestinal inflammation. It induces ER stress in the intestinal epithelium and promotes the excessive activation of the cGAS-STING pathway in the absence of ATG16L1. This leads to type I interferon production and cell death [30]. Based on these observations, it may be tempting to conclude that IL-17, which is produced by Th17 cells, plays a significant role in causing IBD. However, the extent of its involvement in the development of IBD remains a matter of debate. In the proper environment, Th17 cells can transform into a phenotype that more closely resembles Th1 cells, which is characterized by the production of IFNγ [56,57]. IFNγ-producing Th17 cells, which are enhanced by IL-23, may play a crucial role as a pathogenic cell type in IBD. An increase in IFNγ+ IL-17A+ CD4+ T cells has been observed in the inflamed mucosa of individuals with CD and UC in comparison with non-inflamed controls [58,59]. Other significant sources of IFNγ in the inflamed mucosa may include ILCs and natural killer (NK) cells, which are also reactive to IL-23 [60]. IL-12 and IL-23 are cytokines produced primarily by dendritic cells and macrophages. These heterodimeric molecules share a common p40 subunit. The binding of IL-12 and IL-23 to their receptors promotes the expansion and differentiation of Th1 and Th17 cells, respectively. Th17 T cell differentiation, which involves the activation of RORγt, leads to the production of proinflammatory cytokines such as IL-17, IL-6, and tumor necrosis factor (TNF). ILCs and invariant NKT cells, which also respond to IL-23 signaling, create comparable cytokines to Th17 cells. These include IL-17, interferon (IFN)-γ, IL-22, and GM-CSF [61]. Interleukin-12p70 (IL-12p70) induces T-helper-1-cell responses. IL-23, a related cytokine, plays a central role in several T-cell-mediated inflammatory disorders. IL-23 binds to a receptor complex called IL-23R, which is made up of the β1 subunit of IL-12 (IL-12Rβ1) and a subunit specific to IL-23 (IL-23Rα) [62]. The IL-23 receptor complex consists of IL-12Rβ1 and IL-23R transmembrane proteins, both of which have strong binding abilities for the p40 and p19 cytokine subunits [63]. However, it is still unclear how exactly IL-23 and IL-12 responses in the intestine are related. They share a significant ability to stimulate pathogenic Th1 responses, which suggests important overlaps between the two. Therefore, both IL-12-dependent and IL-23-dependent mechanisms could play a role in intestinal pathology and might even work together to produce the most potent inflammatory responses [22]. IL-23-activated Th17 cells produce a range of cytokines, including tumor necrosis factor-α (TNFα), INFγ, IL-6, IL-17A, IL-17F, IL-21, and IL-22 [64,65]. The IL-17 cytokine family is made up of six proteins, specifically, IL-17A to IL-17F. IL-17A is a proinflammatory cytokine that is produced by various cell types and is widely distributed. Its main function is to amplify the inflammatory response by sustaining the release of other inflammatory mediators such as TNF-α and IL-6 [66]. This is achieved by inducing neutrophil-related genes such as CXC-chemokine ligand 1 (CXCL1), CXCL2, and CXCL5, which are involved in inflammatory processes [22]. When neutrophils become activated, they can lead to a positive feedback loop that produces IL-17A and IL-22 [67]. On the other hand, IL-17A supports the expression of genes that promote antimicrobial activity or improve the integrity of the epithelial barrier, such as REG proteins, S100 proteins, lipocalin 2, lactoferrin, β-defensins, claudin, and zona occludens 1 [68,69]. The RORγt transcription factor plays an important role in the differentiation of Th17 cells by regulating the expression of Th17 genes and the IL-23/IL-17 axis [70]. Upon stimulation with IL-23, the receptor triggers the JAK-STAT signaling pathway, which promotes the production of proinflammatory cytokines. When JAK kinase 2 and tyrosine kinase 2 are activated, they trigger the translocation of the STAT3-STAT4 (signal transducer and activator of transcription) dimer to the nucleus, where it activates gene expression [71]. IL-23 is primarily expressed by CD14+ intestinal macrophages, which are crucial in perpetuating inflammation by infiltrating the inflamed intestine in patients with Crohn’s disease [31,58,72]. This is backed up by a recently published study that indicates mucosal TNFR2-expressing CD4+ T cells are able to avoid anti-TNF-induced apoptosis by co-expressing IL-23R, which is activated by the increased production of IL-23 by mucosal CD14+ macrophages. In the study, IL-23 activated pSTAT3 in CD4+ mucosal T cells, resulting in resistance to apoptotic signals. This activation led to the release of high levels of Th1 and Th17 cytokines by the T cells. These TNFR2+IL23R+T cells expanded and accumulated in the mucosa of CD patients who were unresponsive to anti-TNF treatment, perpetuating chronic intestinal inflammation [58]. Based on this data, it seems that patients who are resistant to anti-TNF treatments may find therapies that specifically target IL-23 to be beneficial. Various studies have shown that, when IL-23 interacts with its receptor, it typically results in the phosphorylation of STAT3. This leads to the creation of a positive feedback loop that triggers the expression of genes that are important for the activation and effector functions of Th17 cells [73].

### 4.2. Innate Lymphoid Cells and the IL-23-IL-22 Axis

IL-23 plays an important role in regulating ILC function. Upon stimulation with IL-23, LC3s release significant levels of IL-17 and IL-22 [74,75,76]. According to reports, IL-22 is dependent on both STAT3 [77] and STAT5 activation [78]. ILC cells that are responsive to IL-23 are more prevalent in the inflamed ilea and colons of Crohn’s disease patients compared with the non-inflamed mucosa of healthy individuals. However, these cells are not increased in ulcerative colitis patients [79]. IL-22 is produced by Th17 cells, γδ T cells, and neutrophilic granulocytes in response to IL-23 [80]. Reg3b is a gene that is targeted by STAT3 and is produced by epithelial cells in response to IL-23. It can act as a chemoattractant, which draws neutrophils to the site, and it ultimately contributes to the production of IL-22 in the mucosa [81]. Th17 cells infiltrate the inflamed intestines of individuals with Crohn’s disease. There, they produce proinflammatory cytokines such as IL-17, which perpetuates the inflammatory process [82]. In addition to Th17 cells, several types of innate immune cells are activated by IL-23, including certain subsets of γδ T cells, natural killer T (NKT) cells, intrathymically primed “natural” Th17 cells, and innate lymphoid cells (ILC) [73]. When Th17 cells are stimulated with IL-1β and IL-23, it can lead to inflammation in the nearby tissues. This inflammation is characterized by Th17 signature cytokines such as IL-17, IL-22, and GM-CSF [64]. ILC3 cells express the transcription factor RORγt and play an important role in protecting against extracellular pathogens in the gastrointestinal mucosa. IL-23-responsive ILCs are present in human-mucosa-associated lymphoid tissue, including intestinal Peyer’s patches [75]. Recent studies have shown that γδ T cells play a crucial role as innate IL-17-producing cells in both autoimmune inflammation and infectious diseases [83,84]. After being stimulated with IL-23, γδ T cells begin to produce IL-22, IL-21, and IL-17. Upon stimulation with IL-23, NKT cells produce significant amounts of IL-22 and IL-17 [55].

### 4.3. Regulatory T Cells

**Figure 3:**
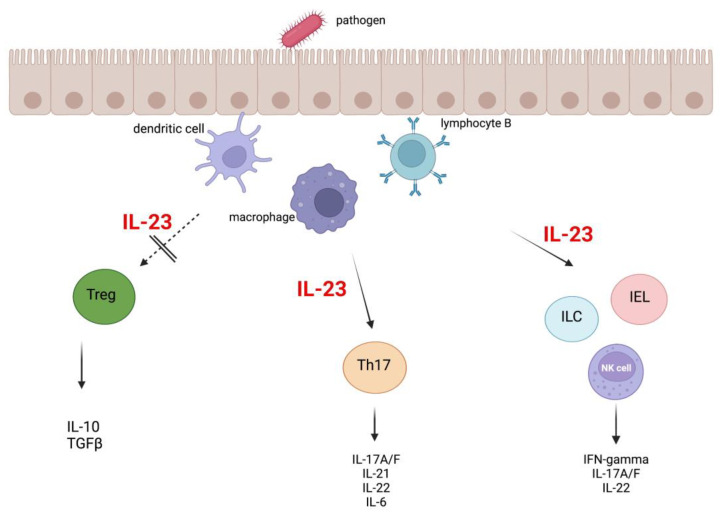
IL-23 controls the activation and cytokine production of immune cells—natural killer (NK) cells, intraepithelial lymphocytes (IEL), innate lymphoid cells (ILCs), and T helper 17 cells (Th17). Additionally, IL-23 blocks the activation of regulatory T cells such as Tr1 and Treg.

A recent study showed that IL-23 may worsen chronic intestinal inflammation by limiting Treg activity in the intestine [85]. According to a previous study, IL-23 may directly hinder T cells’ expression of FoxP3 [86]. In vitro studies indicate that IL-23 does not interfere with the induction of FoxP3 via TGF-β in naive T cells [85]. It is unclear whether IL-23 acts directly or indirectly through other factors to suppress Treg activity in the gut. Tregs are identified by the presence of the FoxP3 transcription factor, high CD25 expression on their cell surface, and the ability to produce TGFβ and anti-inflammatory cytokines such as IL-10 and IL-35 [18,87]. It has been suggested that IL-23 may prevent the development of FoxP3+ CD4+ Tregs in the intestine [85] via cell-intrinsic mechanisms [88]. Treg cells were found to suppress the production of IL-23 and IL-1β from CX3CR1+ macrophages that reside in the intestine but not from CD103+ dendritic cells. This effect is not related to the production of IL-10 and rather appears to be dependent on cell contact and the expression of the latent activation gene-3 (LAG3). As a result, IL-22 production by ILC3s is inhibited by suppressing the production of IL-23 and IL-1β by CX3CR1+ macrophages [89]. T cell responsiveness to IL-23 is crucial in the development of intestinal disease but not in systemic inflammation. IL-23 directly signals T cells, stimulating their proliferation in the intestine. This increases the number of Th17 cells in the intestine and encourages the development of T cells that produce both IL-17A and IFN-γ. In addition, IL-23R signaling in T cells within the intestine inhibits the development of Foxp3+ cells and the production of IL-10 by T cells. In one study, while IL-23r−/− T cells did not have any issues with Th1 cell differentiation, they did experience problems with proliferation and were not able to build up in the intestine [88]. Based on current evidence, it seems that IL-23 is responsible for causing chronic intestinal inflammation by triggering a variety of inflammatory responses. When considered together, the observations suggest that IL-23 initiates a variety of proinflammatory reactions in the gut, including both immediate innate responses and harmful adaptive responses. If not properly addressed, these reactions can result in the emergence of long-term intestinal inflammation [22]. IL-23 can cause the release of proinflammatory cytokines such as IL-1β, IL-6, and TNF-α from myeloid cells. This can then stimulate the secretion of more proinflammatory mediators by stromal, endothelial, and epithelial cells [25] (Figure 3).

## 5. Myeloid Cells

Neutrophil infiltration of the intestinal mucosa is a crucial characteristic of experimental colitis and inflamed lesions in human inflammatory bowel disease, especially ulcerative colitis [90]. After leukocyte activation, STAT3 translocates to the nucleus and regulates cytokine production. STAT3 is a transcriptional regulator involved in the IL-23 pathway, which is identified as a susceptibility gene for IBD [91]. Neutrophils can continue to cause ongoing inflammation in the intestine by producing proinflammatory cytokines, chemokines, and matrix metalloproteases [90,92,93]. New findings indicate that IL-23 serves as a molecular switch that triggers abnormal STAT3 and RORγt transcription-factor-dependent responses in T cells and ILCs [88,94]. Intestinal inflammation driven by IL-23 leads to significant changes in both hematopoietic progenitor and stem cell compartments, which can directly contribute to the development of the disease. Colitis led to significant increases in hematopoietic stem cell (HSCc) proliferation in the bone marrow in a mouse model. The most notable change was the accumulation of long-term hematopoietic stem cells (LT-HSCs), indicating that inflammation associated with colitis had a substantial impact on the earliest stages of hematopoiesis. These findings suggest that the rise in inflammatory myeloid cells in the colon caused by colitogenic Th1- and Th17-cell-mediated responses may be fueled in part by the accumulation of colonic hematopoietic stem and progenitor cells (HSPCs) [93]. IFN-γ may enhance hematopoietic activity at the level of primordial HSCs, which maintain the increased production of downstream and non-self-renewing GMPs. GM-CSF plays a vital role in causing GMPs to accumulate outside the bone marrow in cases of IL-23-dependent intestinal inflammation. This process is a major contributor to the development of the disease. IL-23 enhances the production of GM-CSF by Th17 cells, leading to increased extramedullary hematopoiesis and the buildup of proliferative granulocyte–monocyte progenitor cells in the colon and spleen [93]. IL-23 could potentially enhance the recruitment of neutrophils through both IL-17A-dependent and IL-17A-independent pathways [95,96]. In response to *Clostridioides difficile* infection, IL-23 promotes neutrophil recruitment and the expression of inflammatory cytokines and chemokines in the colon. However, IL-17a and IL-22 do not have this effect [95]. IL-17/IL-17F production is dependent on IL-23 during an acute Mp infection. This production contributes to the recruitment and activity of neutrophils in the lungs, which defend against infection [96]. IL-23 appears to indirectly support the production of granulocytes [97]. The removal of apoptotic neutrophils by DCs through efferocytosis has the potential to regulate neutrophil production. This occurs through the dampening of IL-23 production, which is part of a negative feedback loop. GM-CSF increases the production and activity of eosinophils, which are thought to cause tissue damage in colitis through eosinophil peroxidase. Since murine experimental colitis induced by *Helicobacter hepaticus* and IL-10R blockade improve with the chemical inhibition of eosinophil peroxidase, targeting it could be a promising area for future development [98]. Recent studies have revealed the role of BATF2 in regulating the IL-23/IL-17 pathway in innate myeloid cells. BATF2 is a key molecule for suppressing IL-23 production and preventing T-cell-mediated intestinal inflammation, making it important for regulating the pathogenesis of mucosal inflammation in the intestine [99]. Proinflammatory intestinal macrophages that lack IL-10R produce IL-23, which promotes the accumulation and IL-22 production of both Th17 cells and ILC3. IL-22 is a required factor for colitis initiation, and its induction is the critical colitogenic response to IL-23a produced by IL-10R-deficient proinflammatory macrophages. IL-23 produced by mutant cells emerges as a critical trigger of Th17 cells that secrete IL-22 and thereby initiate an epithelial cell response that results in the deleterious recruitment of neutrophils [100]. In one study, the production of IL-23, which is dependent on TLR8 in human neutrophils, was enhanced by TNF. Additionally, the supernatants from neutrophils stimulated by TLR8 facilitated the differentiation of naïve T cells into Th17 cells [101]. Myeloid cells, including macrophages, express the IL-23R on their cell surface at low levels, suggesting potential autocrine effects. IL-23 triggers the release of cytokines that promote inflammation via macrophages derived from human monocytes. This occurs simultaneously with the activation of IL-23R endocytic recycling and the recruitment of signaling intermediates such as IL-12Rβ1 and JAK2 to the IL-23R complex. Macrophages that were cultured from donors who expressed the protective R381Q IL-23R variant seemed to have a decreased ability to induce cytokines. This was demonstrated by a reduction in IL-23R recycling [102]. During anti-TNF therapy, there is a significant upregulation of mucosal IL-23p19, IL-23R, and IL-17A in anti-TNF refractory patients but not in responders. Apoptosis-resistant TNFR2+IL23R+ T cells expand in anti-TNF refractory patients and perpetuate mucosal inflammation [58].

## 6. IL-23 and IBD

In recent years it has been established that IL-23 is necessary for the development of several autoimmune diseases. It is crucial in psoriatic skin inflammation, experimental autoimmune encephalomyelitis, and inflammatory bowel disease (IBD) [24,28,103]. Early studies associated IL-12p40 solely with chronic inflammatory diseases, which was interpreted as the main role of IL-12-driven Th1 in pathogenesis [104]. IL-23 was first described by Oppmann et al. in 2000, who detected a p19 protein that forms a complex with the IL-12p40 subunit, which gave rise to the biologically active IL-23 protein [28]. The secretion of IL-23 is mainly mediated by antigen-presenting cells in gastrointestinal and respiratory epithelial and secretory cells [105,106]. IL-23 controls the production and activation of numerous different immune cells in the digestive tract by stimulating the IL-23 receptor, which is expressed by several innate and adaptive immune cells, such as natural killer cells (NK), T helper 17 cells (Th17), intraepithelial lymphocytes (IEL), and innate lymphoid cells (ILCs). On the other hand, this cytokine prevents the activation of regulatory T cells (Treg) [23]. Research in the last few years shows the main role of the cytokine IL-23 in the genesis of inflammatory bowel diseases and colon cancer associated with colitis [23].

Serum IL-23 levels have demonstrated greater potential for the diagnosis of IBD than dedicated inflammatory biomarkers such as FCal (fecal calprotectin), CRP (C-reactive protein), and albumin. IL-23 serum level in patients with IBD could differentiate subgroups of patients with severe and non-severe disease. IL-23 and FCal were significantly higher in severe CD and UC compared with mild or moderate; however, the diagnostic role of IL-23 was superior to FCal in discriminating between severe and mild to moderate CD [107]. Research has shown that the serum level of IL-23 is positively correlated with disease duration. It could be suggested that IL-23 is an indicator of prognosis in these patients [108]. According to another study, CD samples demonstrated significant expression levels of IL-17, IL-23, and IL-32 mRNA compared with non-IBD samples, while UC specimens did not. IL-32 is stimulated by Th1 cytokines such as IL-12 and IFN-gamma and appears to strengthen the innate immune response via a nucleotide-binding oligomerization domain protein (NOD2)-dependent pathway [109]. The presented result confirms the overall hypothesis that CD is characterized by a Th1-driven response. The pathways mediated by IL-23 play an important role in the development and expansion of Th17 cells after Th1 activation [109,110]. Furthermore, significantly higher levels of serum IL-6, IL-12, and IL-23 were observed in IBD patients with poor sleep [111]. The conducted research has revealed the elevated expression of IL-23R, IL-23, and IFN-γ in colon mucosa during both acute and chronic colitis [112].

## 7. Intestinal Homeostasis—Mice Models of Inflammatory Bowel Diseases

Key information about the features of IL-12 and IL-23 was obtained from experiments using models mimicking IBD. In healthy mice, the high production of IL-23p19 in the small intestine, with a peak in the terminal ileum, was noted [34]. This was associated with the high expression of p40 mRNA, p40, and IL-23p19/p40 proteins. Dendritic cells CD8alpha and CD11b double-negative CD11c+ lamina propria represent the main passage for IL-12p40, and they are mainly a population of cells in the small intestine in the lamina propria [23,24]. In this area, Th17 cells are present, and they receive immunosuppressive properties, so they are regulatory Th17 cells, which produce cytokines (IL-10, TGF-beta) to protect the mucosa from inflammation [113,114,115]. Research on murine models of autoimmune diseases has demonstrated that IL-23 plays the main role in driving autoimmune tissue pathology [116], and it is associated with the accumulation of a subset of CD4+ T cells secreting IL-17A, which were later defined as Th17 cells [117,118]. In one study, the depletion of IL-23 via the genetic ablation of IL-23p19 or using antibodies led to the attenuation of T-cell-dependent colitis in models of IBD [119] and restained spontaneous colitis in mice, which confirmed the hypothesis that IL-23 plays a role in IBD [120]. An increased level of IL-23 can be observed in different inflammatory bowel diseases (colitis, DSS colitis, TNBS colitis, *Helicobacter hepaticus* colitis, and T cell colitis). These findings emphasized the pivotal role of cytokine in mucosal inflammation [23,120,121,122]. Studies on mice with IL-23 deficiency showed that they developed colitis when their T cells could not respond to the presence of TGF-beta, meaning that, in the colon, TGF-beta may demonstrate proinflammatory properties [6,52,85]. Mice without the expression of IL-23p19 or the p40 subunit are unable to remove *Citrobacter rodentium* after oral administration, leading to death [53]. Furthermore, lacking the expression of the p19 subunit leads to lethal infections and diminishes IL-17 responses in the case of *Klebsiella pneumonia* infections [123]. This confirms that IL-23 plays a crucial role in responses to pathogens and inflammation in organisms. In initial studies, the hypothesis of the role of IL-12p40 in IBD was confirmed by observations on animal models in which genetic ablation using monoclonal antibodies against the IL-12p40 subunit caused the blockage or attenuation of intestinal inflammation [124,125]. Further research has proved the presence of IL-23 is necessary for the induction of intestinal inflammation, as the neutralization of IL-23 blocked the development of colitis in RAG mice and typhlitis, which is caused by *Helicobacter hepaticus23* infections. On the other hand, the neutralization of IL-12 has had no impact on the development of innate immune-mediated or T-cell-dependent inflammation [119,120,126]. The discovery of anti-IL-12p40 antibodies’ ability to neutralize IL-12 and IL-23 promoted a reassessment of their role in chronic inflammatory diseases [22].

## 8. Genetic Correlations

Genome-wide association studies (GWASs) have been performed to investigate pathogenic mechanisms of chronic inflammatory diseases using animal studies. Mice models with spontaneous or induced mutations in several genes revealed internal relationships that may control host responses to inflammation and pathways that lead to chronic inflammatory diseases [127]. The research has included several diseases, such as Crohn’s disease (CD), ulcerative colitis (UC), ankylosing spondylitis (AS), rheumatoid arthritis, and others. These studies explored disease associations with multiple loci that are shared by some chronic inflammatory diseases [128]. The identification of the IL-23 receptor gene as a gene of inflammatory bowel disease provides the first confirmation of the IL-23 pathway’s role in IBD development [129]. It is now well established and confirmed that genetic variants of IL-23 are linked with Crohn’s disease and ulcerative colitis. Genome-wide association studies of Crohn’s disease by the Wellcome Trust Case–Control Consortium identified biological pathways responsible for IBD pathogenesis. They showed genes related to CD risk, IRGM, ATG16L1, NOD2, and IL-23R, which strongly implicate defects, particularly in innate immune responses [130,131,132]. GWASs have identified the polymorphism of the IL-23R gene, encoding the specific receptor of IL-23. Some variants can contribute to IBD but may also protect against the disease. The IL-23R variants are placed in various domains of the folded receptor. R381Q is in the C-terminal cytoplasmic portion, V362I is in the transmembrane region, and G149R is in the extracellular part of the receptor. Additionally, R381Q and V362I variants have inferior protein stability that can lead to lower expression levels, but the G149R variant is in the endoplasmic reticulum as unfolded polypeptides [133]. The R381Q protective variant was identified by a GWAS [134], while G149R and V362I were identified via sequencing [135]. In the case of all protective variants of IL-23R, there is decreased cell surface expression in the receptors because of reduced stability or impaired transferring from the ER. Reduced surface expression causes reduced IL-23-mediated signaling. For example, the most important variant, p.Arg381Gln—consisting of arginine substituted for glutamine at position 381 in the cytoplasmic tail of IL-23R—plays a role in protecting against developing IBD. The presence of glutamine at this position diminishes the stability of the IL-23R protein, which reduces receptor expression at the top of the cells [129,136,137]. IL-23R is expressed in activated T cells; natural killer (NK) cells; and monocytes, macrophages, and dendritic cells [25]. In summary, three variants located at distinct regions of the receptor can be expressed at lower levels at the top of the plasma membrane through different mechanisms, which results in IL-23R-mediated signaling and plays a protective role in IBD [133]. Furthermore, IL-23 is a strong factor that promotes tumor development, and it is at a higher level in human colorectal tumors [138]. This suggests that therapies against IL-23 may be potential prophylactic treatments for colon cancer in patients with IBD. The strategy of treatment should consider the gene polymorphism and genotype of IL-23R [139]. Gheita et al. detected a significantly higher level of IL-23 in IBD patients compared with healthy people. The levels were higher in CD patients compared with those with UC. Furthermore, it was especially high in patients suffering from IBD-associated peripheral and/or axial arthritis [140]. The above results emphasize the critical role of IL-23 in the pathogenesis of inflammation in the intestine and provide important new therapeutic possibilities.

## 9. Treatment

IL-23 and IL-12 are cytokines that cause inflammation and encourage naive CD4+ precursor T cells to develop into T-helper 1 and T-helper 17 cells. Blocking these cytokines has recently become a promising treatment option for inflammatory bowel disease [64]. The IL-23 and IL-12 pathways overlap as they share the cytokine subunit (p40) and a subunit of their receptors (IL-12Rβ1) [141].

**Figure 4:**
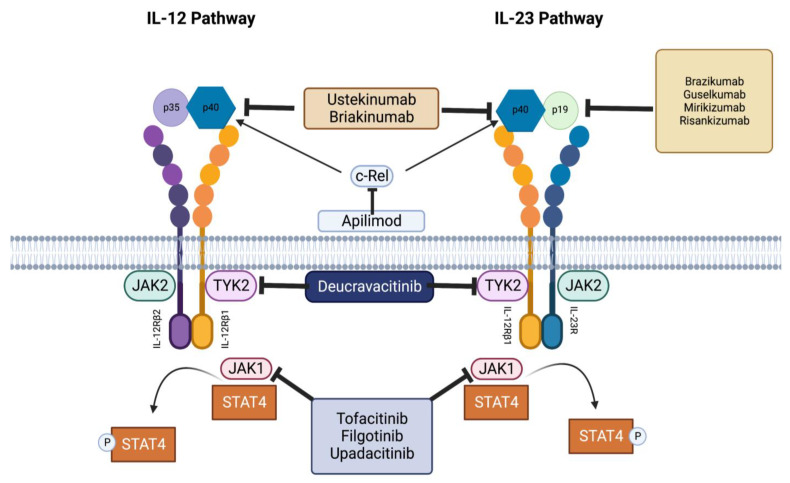
Molecular targets of anti-IL-23 therapies—first, second, and third generation. There is increasing interest in understanding the underlying molecular mechanisms that guide these therapies. Researchers are exploring a wide range of targets, including cytokine receptors, Janus kinases (JAKs), signal transducers, enzymes (TYK2—Tyrosine kinase2), activators of transcription (STATs), and various other intracellular signaling pathways. As such, there is a pressing need for continued research in this area in order to fully understand the molecular targets of anti-IL-23 therapies and develop more effective treatments for patients with a wide range of inflammatory diseases.

Modern methods of treating IBD that target IL-23, both currently used and emerging, can be classified into three generations [142] (Figure 4).

### 9.1. The First Generation

The first-generation treatment involves a therapy that targets anti-IL-12p40. It is important to note that the effectiveness of this treatment is believed to come from inhibiting IL-23 rather than blocking IL-12. The primary representative of the first generation is ustekinumab, a fully human monoclonal antibody that targets the p40 subunit of IL-12 and IL-23 [143], which has been approved for inducing and maintaining clinical responses, as well as achieving remission in patients with moderate-to-severe Crohn’s disease who have previously failed anti-TNF-a therapy [144]. Briakinumab is another type of anti-p40 antibody that has been shown to be effective in treating psoriasis. While it was found to have higher response rates in clinical trials for Crohn’s disease, the study’s primary objective was not achieved [145]. In addition, apilimod has been shown to inhibit the nuclear accumulation of c-Rel [146], the transcription factor that was found to regulate the expression of the IL-12/23 p40 subunit, the IL-23p19 subunit, and the IL-12p35 subunit [147,148,149,150].

### 9.2. The Second Generation

The second generation of selective anti-IL-23 therapies targets IL-23p19 and has demonstrated promising results in other immune-mediated inflammatory disorders, as well as in ongoing clinical trials with IBD patients. Inflammatory bowel disease has been evaluated with four different second-generation anti-IL-23 therapies, namely, brazikumab, guselkumab, mirikizumab, and risankizumab. Brazikumab is a type of monoclonal antibody that targets the p19 subunit of IL-23 but not IL-12. In clinical trials, it was found to be effective in 49% of patients with CD who did not respond to TNF treatments [151]. In a recent study, it was found that risankizumab, an anti-p19 antibody with selective activity against the IL-23 antibody, resulted in remission for 31% of treated CD patients [152]. Furthermore, it appears that mirikizumab is effective in both inducing and maintaining remission in patients with UC [153,154] and CD [155]. Guselkumab and tildrakizumab appear to be promising antibodies against p19 [156], and studies of guselkumab in IBD are ongoing. Most drugs that inhibit IL-12/IL-23 were first tested on CD. However, when it comes to treating UC with p40 or p19 inhibition, experience is more limited, with the exception of mirikizumab and ustekinumab.

### 9.3. The Third Generation

The third generation refers to a group of small molecule inhibitors that target signaling molecules activated downstream of IL-23R, specifically, Janus kinase inhibitors. Considering the significance of the JAK/STAT pathway in IBD pathogenesis, a new category of SMDs (small-molecule drugs) called JAK inhibitors is currently under development [157]. Tofacitinib selectively inhibits JAK1 and JAK3. In clinical trials for phases II and III, it was demonstrated to be a safe and effective option for induction and maintenance therapy in UC but not in CD [158,159,160,161,162]. In a phase II evaluation, two JAK inhibitors, filgotinib and upadacitinib, demonstrated promising outcomes for clinical and endoscopic remission in patients with Crohn’s disease and ulcerative colitis. Filgotinib is currently being evaluated in phase III studies [163]. Moreover, filgotinib and updacitinib have been approved for the treatment of moderate-to-severe UC [164,165].

A phase II trial is currently being conducted, a randomized, double-blinded, placebo-controlled study of deucravacitinib, an oral selective TYK2 inhibitor. The study aims to determine the safety, efficacy, and biomarker response of the drug in subjects with moderate-to-severe UC and CD [166,167,168].

### 9.4. Results of Treatment

Information about the meaning of IL-23 signaling in inflammation can be derived from animal model work, and currently, it may be used to develop treatments for inflammatory bowel diseases [73]. In the past, the approach to IBD was empiric, and it preferred new techniques at different times. Studies led to cell and cytokine identifications; genome and bacterial genome analysis opened up new ways of understanding inflammation and pathogenic mechanisms, giving way to new treatment opportunities. The first-generation anti-IL-23 therapy ustekinumab (anti-IL-12p40 monoclonal antibody) is secure and effective, which can be seen in CD and UC trials [169,170]. The comparison of adalimumab with ustekinumab suggests better tolerance of the second, but there is no difference between them in any major point [171]. Second- and third-generation inhibitors (brazikumab, guselkumab, mirikizumab, risakizumab, and deucravacitinib), which are selective only for the IL-23 pathway, are now in development and show promising safety and efficacy in trials [151,172]. Notwithstanding, successful drug development remains open-ended because the aim of IBD treatment should be a cure, not just improving the clinical condition of patients and slowing down the progression of the disease.

## 10. Conclusions and Future Perspectives

The cytokine networks involved in the pathogenesis of inflammatory bowel disease (IBD) are highly complex. The findings presented above clearly demonstrate the crucial role of the cytokine IL-23 in the development of IBD.

IL-23 undoubtedly plays a crucial role in the development of inflammatory bowel disease (IBD) by influencing mucosal immunity. Research has indicated that IL-23 is particularly vital in sustaining and increasing the Th17 lineage through a positive feedback loop that enhances levels of IL-17, RORγt, TNF, IL-1, and IL-6. However, the mechanisms by which IL-23 drives chronic IBD remain a matter of debate and investigation. Specifically, the relative importance of Th1/Th17 cells, ILC3s, and myeloid cells, as well as the cytokines IFNγ, IL-22, and IL-17A in colitogenesis, are under discussion. The discovery of the IL-23/IL-17 axis has provided new insights into the pathology of chronic inflammatory diseases such as IBD and has revealed a novel way in which immune responses can trigger tissue damage in the intestine.

Targeting IL-23 has become an important concept in suppressing gut inflammation. Neutralizing antibodies against IL-12/IL-23p40 have been approved for therapy for Crohn’s disease. IL-23p19-specific blockers have shown promising results in other immune-mediated inflammatory disorders, as well as in ongoing clinical trials on patients with inflammatory bowel disease (IBD). In addition, small-molecule drugs (SMDs) that target signaling molecules activated downstream of the IL-23R are currently being developed.

Based on the presented data, it has been observed that, in recent times, a number of monoclonal antibodies that specifically target IL-23 p19, such as risankizumab, mirikizumab, and brazikumab, have demonstrated both efficacy and safety in treating patients with CD or UC. 

According to current in-progress studies, subcutaneous risankizumab is a safe and effective treatment for maintaining remission in patients with moderately to severely active Crohn’s disease. This new therapeutic option offers hope to a wide range of patients by meeting endpoints that could potentially impact the future course of the disease [173].

The results of the studies conducted during the induction and maintenance phase 3 trials demonstrate that the use of mirikizumab effectively reduces bowel urgency in patients who suffer from moderately to severely active ulcerative colitis [174]. Additionally, it has been shown to improve patients’ health-related quality of life (HRQoL). Further trials assessing the efficacy and pharmacoeconomic benefits of mirikizumab are currently ongoing and will provide future data [175].

In a phase 2a trial, patients with moderate-to-severe Crohn’s disease who failed treatment with tumor necrosis factor antagonists showed clinical improvement when treated with brazikumab [151]. Currently, a phase 2b/3 program is being conducted to assess the effectiveness of brazikumab compared with placebos or adalimumab in CD [176].

Targeting the JAK/STAT pathway and proinflammatory cytokines may offer a promising treatment for IBD, particularly for those who have not responded to other therapies. JAK inhibitors have demonstrated good results in treating moderate-to-severe UC and CD. They may be a viable alternative to biologic therapies, as they are orally administered and act quickly without provoking any immune reactions. Nonetheless, there are still concerns about their safety, such as the risk of infections, blood clots, and long-term effects. Further research is required to ascertain the risks and benefits of JAK inhibitors and their place in the treatment of IBD [177].

The development of vaccines targeting cytokines as key players in inflammation initiation and progression has garnered significant attention. As a potential treatment option for IBD, mRNA vaccines that specifically target the IL-23p19 subunit are currently being explored. The goal of these vaccines is to initiate an immune response against IL-23, effectively reducing its activity and dampening the inflammatory response. In vitro studies have shown that serum IL-23 p19-specific IgG significantly suppresses IL-23-induced IL-17 production by splenocytes. In vivo evaluations of the vaccine’s effect on mice with chronic colitis indicated that vaccine-immunized mice exhibited a decrease in colon inflammation, collagen deposition, and levels of IL-23 and IL-12 cytokines compared with the control groups [178,179]. It appears possible to develop a vaccine that would help control immune responses in this inflammatory disease. However, such a vaccine could have side effects and may increase the patient’s risk of opportunistic infections.

Therefore, therapeutic approaches targeting IL-23 inhibition may be particularly important for the treatment of IBD patients in the future. Additional research on molecular and clinical factors that influence responses to these drugs could help identify patients who are most likely to benefit from them.

Given that multiple pathways leading to inflammation are activated in the inflamed intestine, targeting just one pathway may not be sufficient to control the inflammation, as currently happens with targeted monotherapies. Consequently, in the future, we will need to develop treatment strategies, such as sequential or combination therapy, to optimize the efficacy of each drug. The availability of multiple biologics for treating IBDs allows for the possibility of combining them to simultaneously block different pathways, which could lead to additive or synergistic effects for refractory diseases. However, this combination therapy may face certain potential issues, such as blocking opposing pathways, which may result in increased side effects.

To continue advancing the treatment of IBD, it is important to consider exploring alternative methods for administering medication. IL-23 inhibitors can be administered through intravenous infusions. Creating more subcutaneous or oral formulations for these drugs could offer patients more convenient and accessible treatment options, reducing the necessity of hospital visits and enabling self-administration.

Precision medicine is a field that seeks to personalize treatments based on individual patient characteristics. By identifying genetic profiles and biomarkers, we may be able to pinpoint patients who are more likely to respond to IL-23 inhibitors. This can lead to more effective and personalized treatment strategies.

In the pursuit of personalized and precise therapy, there are both opportunities and challenges. Indications, contraindications, and evidence-based medicine for various drugs and treatments should be fully considered to develop individualized treatment plans based on a comprehensive patient assessment. All of the above factors play a necessary role in inducing and maintaining remission in patients with IBD.

## Data Availability

Data sharing is not applicable as no datasets were generated or analyzed during the current study.
